# Role of Macrophage Migration Inhibitory Factor in Obesity, Insulin Resistance, Type 2 Diabetes, and Associated Hepatic Co-Morbidities: A Comprehensive Review of Human and Rodent Studies

**DOI:** 10.3389/fimmu.2015.00308

**Published:** 2015-06-15

**Authors:** Martine C. Morrison, Robert Kleemann

**Affiliations:** ^1^Department of Metabolic Health Research, Netherlands Organization for Applied Scientific Research (TNO), Leiden, Netherlands; ^2^Department of Human and Animal Physiology, Wageningen University, Wageningen, Netherlands

**Keywords:** MIF, obesity, adipose tissue, insulin resistance, type 2 diabetes, non-alcoholic fatty liver disease

## Abstract

Obesity is associated with a chronic low-grade inflammatory state that drives the ­development of obesity-related co-morbidities such as insulin resistance/type 2 diabetes, non-alcoholic fatty liver disease (NAFLD), and cardiovascular disease. This metabolic inflammation is thought to originate in the adipose tissue, which becomes inflamed and insulin resistant when it is no longer able to expand in response to excess caloric and nutrient intake. The production of inflammatory mediators by dysfunctional adipose tissue is thought to drive the development of more complex forms of disease such as type 2 diabetes and NAFLD. An important factor that may contribute to metabolic inflammation is the cytokine macrophage migration inhibitory factor (MIF). Increasing evidence suggests that MIF is released by adipose tissue in obesity and that it is also involved in metabolic and inflammatory processes that underlie the development of obesity-related pathologies. This review provides a comprehensive summary of our current knowledge on the role of MIF in obesity, its production by adipose tissue, and its involvement in the development of insulin resistance, type 2 diabetes, and NAFLD. We discuss the main findings from recent clinical studies in obese subjects and weight-loss intervention studies as well as results from clinical studies in patients with insulin resistance and type 2 diabetes. Furthermore, we summarize findings from experimental disease models studying the contribution of MIF in obesity and insulin resistance, type 2 diabetes, and hepatic lipid accumulation and fibrosis. Although many of the findings support a pro-inflammatory role of MIF in disease development, recent reports also provide indications that MIF may exert protective effects under certain conditions.

## Introduction

**Figure 1:**
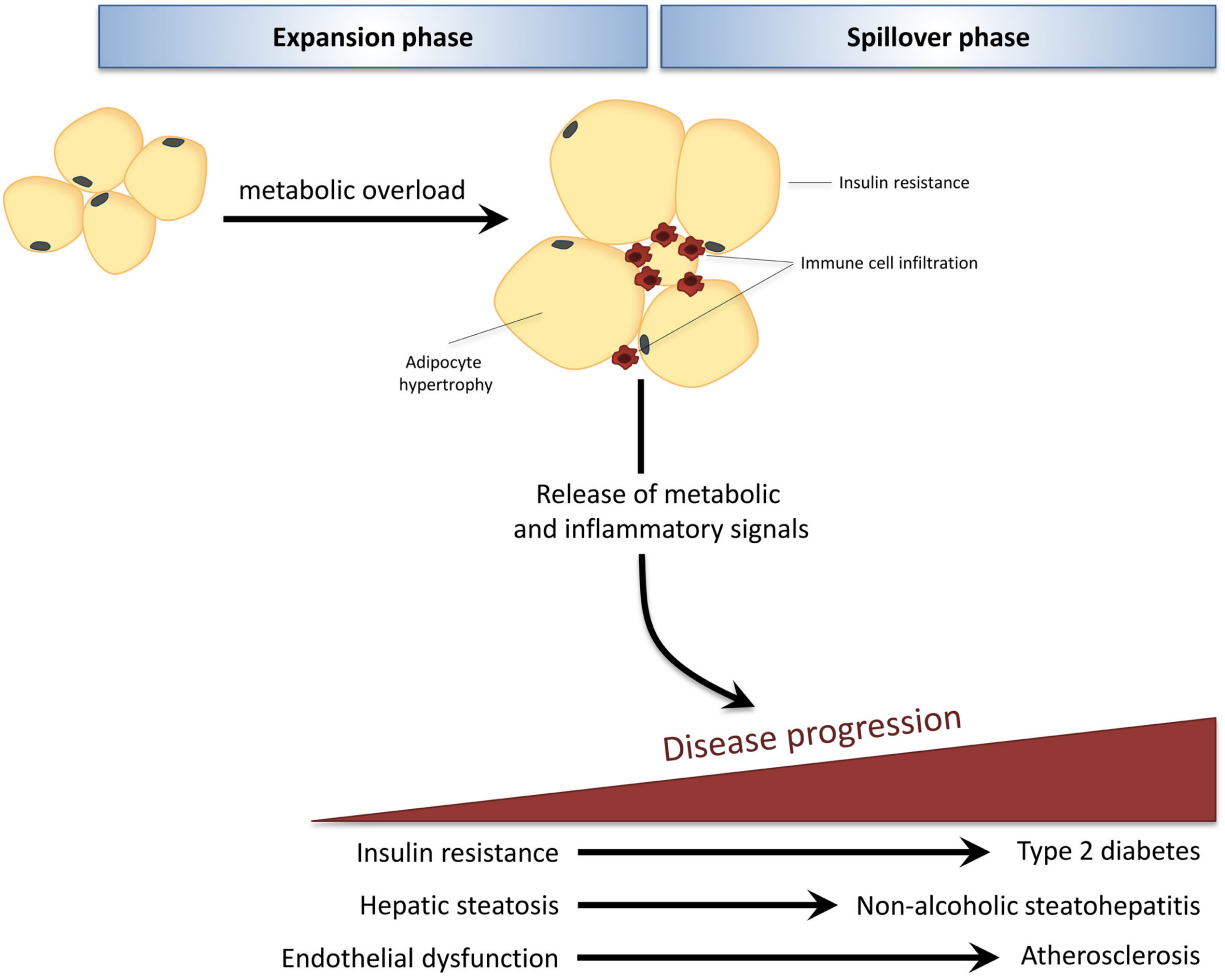
**In obesity, adipose tissue can become a source of inflammation that drives disease development in distant organs**. Caloric and nutrient excess, i.e., metabolic overload, leads to expansion of adipose tissue depots (expansion phase). When the maximal expandability of an adipose tissue depot is reached due to prolonged metabolic overload, the adipose tissue becomes dysfunctional, is infiltrated by immune cells, and becomes insulin resistant. Adipocytes and infiltrated immune cells produce inflammatory mediators that spill over into the circulation (spillover phase) where they drive the progression of obesity-related co-morbidities in distant organs, such as type 2 diabetes, non-alcoholic fatty liver disease, and atherosclerosis.

The development of obesity-associated co-morbidities such as type 2 diabetes (T2D), non-alcoholic fatty liver disease (NAFLD), and cardiovascular disease (CVD) is considered to be driven by the chronic low-grade inflammatory state that characterizes obesity (1). During obesity, excess caloric and nutrient intake, i.e., metabolic overload, leads to adipose tissue expansion and visceral adiposity. When the maximal expandability of an adipose tissue depot is reached (when adipocytes have maximally expanded) this leads to adipose tissue dysfunction and infiltration of immune cells, which is associated with insulin resistance of the adipose tissue itself (2, 3). Furthermore, adipocytes and infiltrated immune cells of this inflamed adipose tissue produce inflammatory mediators that can be released into the circulation (4). Thus, the adipose tissue becomes a source of inflammation that can drive pathogenesis in other tissues, leading to the development of T2D, NAFLD, and CVD (Figure 1).

Macrophage migration inhibitory factor (MIF) is a cytokine that is ubiquitously expressed, both by immune and non-immune cells. It is well known for its pro-inflammatory effects and is recognized as a negative regulator of the immunosuppressive actions of glucocorticoids (5). In line with this, MIF has been implicated in the development of many acute inflammatory and auto-immune diseases (6, 7), as well as chronic inflammatory metabolic disorders (8–10). MIF can act via its receptor CD74 (11), and controls the recruitment of inflammatory cells via CXCR2 and CXCR4 signaling (8). Furthermore, MIF can exert pro-inflammatory effects through its enzymatic tautomerase and oxidoreductase activity (12, 13). Several lines of evidence provide indications that MIF, besides regulating inflammation, may also be linked to energy metabolism. It is expressed in metabolically active tissues such as the adipose tissue and the liver (9, 14). Its expression by adipocytes is regulated by glucose and insulin (15) and it has been shown to have catabolic effects in muscle (16). Furthermore, it co-localizes with insulin within the secretory granules of pancreatic beta cells and is a modulator of insulin release (17). Increasing evidence suggests that MIF may also control inflammatory and metabolic processes in the pathogenesis of obesity and associated disorders including insulin resistance, T2D, and NAFLD. Here, we provide a comprehensive review of the current knowledge on the role of MIF in obesity, MIF production by adipose tissue, and its role in the development of insulin resistance, type 2 diabetes, and NAFLD. Since epidemiological studies have shown both insulin resistance (18, 19) and NAFLD (20, 21) to be associated with future CVD risk, this may also have relevance for the role of MIF in vascular disease, which has been described elsewhere for rodents (22, 23) and humans (24, 25), and will not be addressed here. This survey aims to advance our understanding on the functions of MIF in non-pathological and pathological processes connected to obesity and glucose homeostasis, with particular emphasis on adipose tissue and liver.

All values are mean ± SEM. Where the original article stated mean ± SD, SEM was calculated from SD by dividing SD by the square root of *n*.

## Human Studies

### Circulating MIF and obesity

Several studies provide evidence for a positive association between obesity and circulating MIF levels (Table 1). Dandona and co-workers (26) reported that obese individuals (BMI 37.5 ± 4.9 kg/m^2^) had significantly higher plasma MIF levels (2.8 ± 2.0 ng/ml) than lean individuals (BMI 22.6 ± 3.4 kg/m^2^; plasma MIF 1.2 ± 0.6 ng/ml) and that there was a highly significant positive correlation between MIF levels and BMI. Results from another study by the same group (27) confirmed these results, reporting MIF levels of 3.3 ± 2.4 ng/ml in obese subjects (BMI 40.0 ± 4.4 kg/m^2^), which were significantly higher than the 1.3 ± 0.8 ng/ml observed in the lean controls (BMI 22.6 ± 1.9 kg/m^2^). Again, there was a positive correlation (*p* = 0.10) between MIF levels and BMI. Others have reported similar findings of increased circulating MIF in obese individuals compared with healthy lean controls (28–31), and showed associations with % body fat and % truncal fat (31). Studies in pre-pubescent schoolchildren (age 5–13 years) and male adolescents (age 13–17 years) demonstrate that these associations are not only observed in adults, reporting increased MIF levels in overweight and obesity in childhood and adolescence (32, 33), and showing positive correlations with waist circumference and BMI (33) in this study population as well.

**Table 1:** **Relationship between (circulating) MIF and obesity and effects of weight loss thereupon**.

Subjects	BMI	Gender		MIF (ng/ml)	Effect observed	Reference
		M	F			
Lean	22.6	26	14	1.2	Positive correlation plasma MIF with BMI. MNC *MIF* mRNA increased in obesity and positively correlated with BMI.	(26)
Obese	37.5	19	21	2.8		

Lean	22.6	–	16	1.3	Positive correlation plasma MIF with HOMA and BMI (*p* = 0.10). MNC *MIF* mRNA increased in obesity and positively correlated with BMI.	(27)
Obese	40.0	–	16	3.3		

Lean	20.6	–	20	0.5	Plasma MIF increased in obesity.	(30)
Obese	35.2	–	26	1.9		

Lean	22.1	–	6	5	Plasma MIF positively correlated with % body fat, % truncal fat, and fasting insulin.	(31)
Obese	35.8	–	6	16		

Lean boys	16.0	59	–	3.8	Plasma MIF higher in obese boys than in overweight boys. No effect in girls (population: pre-pubertal schoolchildren between 5 and 13 years, average 9 years).	(32)
Overweight boys	22.0	32	–	3.6		
Obese boys	25.7	70	–	4.2		
Lean girls	15.1	–	46	3.9		
Overweight girls	21.3	–	28	3.9		
Obese girls	25.1	–	70	4.3		

Waist circumference <90th percentile	20.7	41	–	Median 0.6	Positive correlation plasma MIF with weight, BMI, and waist circumference (population: Caucasian adolescents 13–17 years).	(33)
Waist circumference >90th percentile	31.9	38	–	Median 1.0		

Healthy controls	20.2	52	32	1.0	Plasma MIF increased in metabolic syndrome. No significant correlation with BMI.	(34)
Metabolic syndrome	27.2	62	26	1.4		

Lean	23.0	–	14	12.0	No difference in plasma MIF between lean and obese subjects.	(35)
Obese	32.7	–	33	13.5		

Lean	<27	–	ns	nd	MIF secretion from isolated (subcutaneous and omental) adipocytes is positively correlated with BMI. No difference between depots.	(38)
Obese	37	–	ns	nd		

Lean	23.1	9	–	nd	Subcutaneous abdominal adipose tissue *MIF* mRNA expression is increased in obesity and is positively correlated with waist circumference.	(39)
Obese	34.7	9	–	nd		

Overweight	29.3	–	17	nd	*MIF* mRNA expression in visceral adipose tissue twofold higher than in subcutaneous adipose tissue, and positively correlated with % body fat.	(40)

Obese	36	18	16	ns	Subcutaneous abdominal adipocyte *MIF* mRNA expression positively correlated with adipocyte diameter but not with plasma MIF.	(43)

Lean	24.2	21	4	Median 5.1	Plasma MIF higher in obese than in lean subjects. Weight loss (by diet and physical activity based weight management program) reduced plasma MIF levels.	(28)
Obese before weight loss	43.0	23	48	Median 8.4		
Obese after weight loss	38.3			Median 5.1		

Lean	19.9	–	10	5.0	Plasma MIF and mononuclear cell *MIF* mRNA are higher in obese subjects than in lean subjects and both are reduced by weight loss (12-week caloric restriction and light exercise-based weight loss program). Mononuclear cell *MIF* mRNA positively correlated with BMI.	(29)
Obese before weight loss	32.5	–	21	16.0		
Obese after weight loss	30.6			5.4		

Before intervention	27.6			nd	Weight loss did not affect subcutaneous adipose tissue *MIF* mRNA expression.	(44)
Control	27.3	5	6			
Caloric restriction	25.0	6	6			
Caloric restriction + exercise	24.8	5	7			

Obese before surgery	44.6	5	29	0.2	Serum MIF reduced 12 months after bariatric surgery. Positive correlation between reduction in serum MIF and body weight loss.	(45)
Obese after weight loss	35.2			0.02		

Obese before surgery	46.7	5	22	0.2	Plasma MIF levels increased after weight loss (at 24 months after bariatric surgery).	(46)
Obese after weight loss	33.0			0.7		

*ns, not specified; nd, not determined*.

A study by Kim et al. (34) compared healthy Korean subjects (BMI 20.2 kg/m^2^) without metabolic syndrome with patients with metabolic syndrome (BMI 27.2 kg/m^2^) and found higher levels in patients than controls (1.4 ± 0.1 and 1.0 ± 0.1 ng/ml, respectively), which was significant in women but not in men. N.B. there was no correlation between circulating MIF and BMI in this study, suggesting that other MIF-inducing factors play a role when overweight/obesity progresses toward a phenotype of metabolic disease.

Of note, plasma MIF concentrations tend to be higher in males than in females (26, 34), suggesting an inducing effect of male sex hormones. Indeed, circulating MIF levels are two to threefold higher in women with polycystic ovary syndrome (PCOS), an endocrine disorder characterized by elevated levels of androgens (31, 35). Within PCOS patients, the effect of obesity is not ­significant due to large variation within this population, and comparable plasma MIF levels were found in non-obese and obese patients [~35 and ~55 ng/ml, respectively, in Ref. (31); 35.2 and 48.6 ng/ml, respectively, in Ref. (35)].

Remarkably, there is a large variation in the absolute circulating MIF values in documented obesity studies, indicating that there may be underlying methodological differences causing these diverse values. A part of the variation may be explained by different analytical methods (e.g., different ELISA kits, multiplex technology) but even studies employing the same analytical tools report substantial differences [e.g., Ref. (26, 31)]. Hence, other technical or methodological issues may be responsible for these discrepancies. The MIF molecule itself and its oligomers are very hydrophobic (36, 37) and tend to precipitate or stick to plastic material and differences in sample preparation and/or pipetting can result in differences as reported in the human studies. Therefore, it is difficult to make comparisons between different studies. However, the direction of the effect (obese higher than lean) is mostly consistent and overall, these studies demonstrate a well-established association between obesity and circulating MIF that seems to transcend differences in age, sex, and ethnicity. The observed positive correlations with BMI and % body fat provide indication that the observed association may be related to the increased adipose tissue mass that is inherent to obesity and hint at the adipose tissue as a contributor to the observed association.

### Adipose tissue MIF production

Several groups have investigated adipose tissue as a potential source of circulating MIF in obesity (Table 1). Skurk and co-workers (38) demonstrated that primary human pre-adipocytes and mature adipocytes have the capacity to produce and secrete MIF and the secretion of MIF was found to increase in parallel with the (*in vitro*) differentiation of pre-adipocytes. There was a close positive correlation between MIF release from mature adipocytes and donor BMI, which was observed in both visceral and omental adipocytes, with no differences between these two depots. Others have shown similar effects on the gene expression level. González-Muniesa and colleagues (39) showed that *MIF* mRNA expression in subcutaneous abdominal adipose tissue is increased in obese individuals and correlates with waist circumference. A positive correlation was also reported by Alvehus and co-workers (40) for the visceral adipose tissue depot, which is an important source of inflammation and, in contrast to subcutaneous adipose tissue, is associated with inflammatory metabolic disorders such as NAFLD and CVD (41, 42). Alvehus et al. (40) showed that *MIF* mRNA expression in the visceral adipose tissue was higher than in the subcutaneous adipose tissue (~2-fold) and was positively correlated with body fat percentage. Adipose tissue inflammation is considered to be a consequence of adipocyte hypertrophy and is thought to be triggered by maximal expansion of adipocytes (2, 3). In line with this notion, subcutaneous abdominal adipocyte diameter correlates with *MIF* mRNA expression (43).

If indeed the increased levels of circulating MIF in obesity are primarily the result of increased adipose tissue mass and ­adipocyte expansion, one would logically expect plasma MIF levels to decrease when obese subjects lose weight. Indeed, reduced MIF levels have been reported upon weight loss. Church and colleagues (28), for instance, observed significant reductions in plasma MIF levels in obese individuals after a diet and physical activity based weight-loss program. Participants’ BMI after this 8.5-month program was reduced from 43.0 ± 8.6 to 38.3 ± 7.6 kg/m^2^ (reflecting an average weight loss of 14.4 kg), which resulted in completely normalized plasma MIF levels comparable to those of lean controls (from median 8.4 to 5.1 ng/ml). Similar results were reported in a different study on the effects of weight loss (29) in which obese subjects participated in a 3.5-month diet and light exercise-induced weight-loss program. A reduction in BMI from 32.5 ± 1.2 to BMI 30.6 ± 1.6 kg/m^2^ (reflecting a weight loss of 4.0 ± 0.4 kg) was accompanied by a reduction in serum MIF from 16.0 ± 4.0 to 5.4 ± 0.4 ng/ml, which again was comparable to lean control subjects. Remarkably, the observed weight loss in both these studies in obese subjects resulted in completely normalized MIF levels comparable to those observed in lean subjects, even though BMI was still considerably higher in the obese subjects (and subjects were still obese even after weight loss), thus indicating that other factors rather than fat mass *per se* (e.g., insulin sensitivity, hormonal changes) may determine circulating MIF levels, or that weight loss may normalize the state of a particular adipose tissue depot that is primarily responsible for MIF production and secretion. Furthermore, a relatively small reduction in adipocyte size may already be sufficient to prevent further adipocyte damage and thereby attenuate the inflammatory process in adipose tissue.

Only a few studies have examined whether a specific adipose tissue depot is responsible for the release of MIF into the circulation. Support for the view that circulating MIF must be produced by adipose tissue depots other than the subcutaneous adipose tissue comes from studies by Koska et al. (43) who showed that subcutaneous adipose tissue *MIF* mRNA does not correlate with circulating MIF levels, and Tam et al. (44) who found no effect of weight loss on subcutaneous adipose tissue *MIF* mRNA expression. Identification of the underlying factors that determine circulating MIF in obesity warrants further investigation.

Two studies on the effects of bariatric surgery-induced weight loss in morbidly obese subjects report conflicting results in this specific population. Fenske et al. (45) showed that weight loss at 12 months after bariatric surgery (BMI from 44.6 ± 0.9 to 35.2 kg/m^2^) resulted in reductions in serum MIF levels from 0.2 ng/ml before surgery to 0.02 ng/ml at 12 months after surgery. Van Dielen and colleagues (46), on the other hand, reported similarly low-plasma MIF levels in ­morbidly obese subjects before surgery (BMI 46.7 ± 1.1 kg/m^2^, plasma MIF 0.2 ng/ml) but observed increased MIF levels at 24 months after bariatric surgery (weight loss to BMI 33.0 ± 0.9 kg/m^2^, plasma MIF 0.7 ng/ml). To gain insight into the processes that determine circulating MIF levels in these patients, longitudinal analyses after bariatric surgery are needed and it is possible that co-variables such as impaired insulin secretion and diabetes may determine plasma MIF levels in this extreme group of metabolically deregulated subjects.

Overall, the above studies indicate that whole-body adiposity is not a key determinant of circulating MIF and suggest that MIF is produced by specific adipose tissue depots during a particular period of depot growth (i.e., adipocyte expansion within the depot) and resulting adipose tissue inflammation. Of note, circulating MIF may also be produced by other tissues and cells in obesity. Besides adipose tissue as a possible source of circulating MIF in obesity, there are some reports on MIF expression by mononuclear cells (MNCs), with reports of increased *MIF* mRNA expression by MNC from obese subjects (26, 27, 29). Although MNC *MIF* expression did not correlate with plasma MIF levels (26), a correlation with BMI was reported in two of these studies (26, 27) and weight loss (BMI from 32.5 ± 1.2 to 30.6 ± 1.6 kg/m^2^) significantly reduced MNC *MIF* mRNA expression (29).

### Relationship (circulating) MIF and insulin resistance

Results from observational studies have shown increased levels of circulating MIF in insulin resistant and T2D subjects (Table 2). Yabunaka et al. (47) reported increased serum MIF levels in Japanese type 2 diabetic subjects (20.7 ± 1.5 ng/ml) compared with healthy control subjects (5.2 ± 0.3 ng/ml); however, there was no correlation between MIF and fasting plasma glucose, HbA1c, or diabetes duration. Interestingly, BMI was comparable between T2D patients and controls in this study (23.9 ± 0.3 kg/m^2^ in T2D vs. 23.8 ± 0.4 kg/m^2^ in controls) indicating that the observed difference was not attributable to a higher BMI in the diabetic subjects. (N.B. as the study was conducted in an Asian population absolute BMI values are lower than in Caucasian diabetes patients.) Similarly, Yu and colleagues (48) showed increased MIF levels in Chinese T2D patients (2.6 ± 0.1 ng/ml) compared with healthy controls (2.1 ± 0.1 ng/ml), which again were independent of BMI (23.9 ± 0.1 kg/m^2^ in T2D vs. 22.9 ± 0.3 kg/m^2^ in controls). In line with these findings, Sanchez-Zamora et al. reported increased MIF levels (~0.2 vs. ~0.05 ng/ml) in Mexican T2D patients relative to healthy controls (49), although BMI for the different groups was not stated in this study.

**Table 2:** **Relationship between (plasma) MIF and insulin resistance/T2D**.

Subjects	Gender		MIF (ng/ml)	Effect observed	Reference
	M	F			
Healthy controls	53	26	5.2	Serum MIF higher in T2D than in controls.	(47)
T2D	53	26	20.7		

Healthy controls	30		2.1	Plasma MIF higher in T2D than in healthy controls.	(48)
T2D	46		2.6		

Healthy controls	23	59	0.05	Serum MIF higher in T2D than in healthy controls, in both males and females.	(49)
T2D	27	46	0.2		

Normoglycemic controls	137	99	Median 4.97	Positive association plasma MIF with impaired glucose tolerance and T2D independent of plasma CRP and IL-6.	(50)
Impaired glucose tolerance	130	112	Median 7.95		
T2D	137	107	Median 10.96		

Non-diabetic Caucasians	24		<5.0 in 100%	Plasma MIF higher in Pima Indians and associated with insulin resistance.	(51)
Non-diabetic Pima Indians	28		>5.0 in 39%		

Non-case controls	859	773	Median 17.7	Baseline MIF concentrations higher in subjects that develop T2D than in non-case controls. Women with *MIF* genotype rs1007888CC have increased risk of T2D.	(52)
Cases (incident T2D)	293	209	Median 18.5		

Turkish adults	1093	1157	nd	In men, *MIF* genotype rs755622GC associated with baseline diabetes, and C-allele carriage tends to predict new-onset diabetes. No effect in women.	(53)

Healthy pregnant controls	–	40	5.3	Serum MIF higher in women with gestational diabetes.	(54)
Gestational diabetes	–	43	11.3		

Healthy pregnant controls	–	169	nd	*MIF* genotype rs1007888GG more common in gestational diabetes. GG genotype significantly associated with pre-pregnancy obesity and family history of diabetes, and twofold more frequent in women with metabolic syndrome.	(55)
Gestational diabetes	–	147	nd		

*nd, not determined*.

These associations with MIF have also been reported in earlier stages of T2D development (impaired glucose tolerance, insulin resistance), before overt T2D is present. Herder et al. (50) reported increased serum MIF concentrations in German individuals with impaired glucose tolerance (median MIF 7.95 vs. 4.97 ng/ml in healthy controls) and further increased levels in individuals with T2D (median MIF 10.96 ng/ml), suggesting a gradual increase of MIF with severity of the disease. In this study, BMI was higher in the insulin resistant and T2D subjects than in the healthy controls. In line with these results, a positive association between plasma MIF levels and insulin resistance was shown in a study in Pima Indians (51) and correlations with homeostatic model assessment (HOMA; a method used to quantify insulin resistance) (26, 27) and β-cell dysfunction (28) have also been reported. Furthermore, *MIF* mRNA expression in freshly isolated subcutaneous abdominal adipocytes was found to be negatively associated with donor peripheral and hepatic insulin action in another study in Pima Indians (43).

In a prospective case-cohort study (52), baseline levels of serum MIF were found to be higher in incident T2D cases than in non-case controls (mean follow-up 10.1 ± 0.1 years, only significant in women) and this association between elevated MIF serum levels and T2D risk was stronger in obese than in non-obese women. Furthermore, this study reported significant associations between serum MIF levels in women and *MIF* genotype rs1007888CC, and carriers of this allele had a 1.7-fold increased risk of T2D. A different *MIF* genotype (rs755622GC) was shown to be associated with baseline diabetes in men but not in women in a population-based cohort study in Turkish subjects, and carriage of the C-allele tended to predict new-onset diabetes in this population (53).

MIF has also been implicated in gestational diabetes, with higher serum MIF in women with gestational diabetes (11.2 ± 0.75 ng/ml) than in healthy pregnant controls (5.31 ± 0.64 ng/ml) (54). Furthermore, associations between *MIF* genotype Rs1007888GG and gestational diabetes as well as pre-pregnancy obesity and family history of diabetes have been reported (55). This specific genotype was also found to be twofold more frequent in women with metabolic syndrome (55).

**Figure 2:**
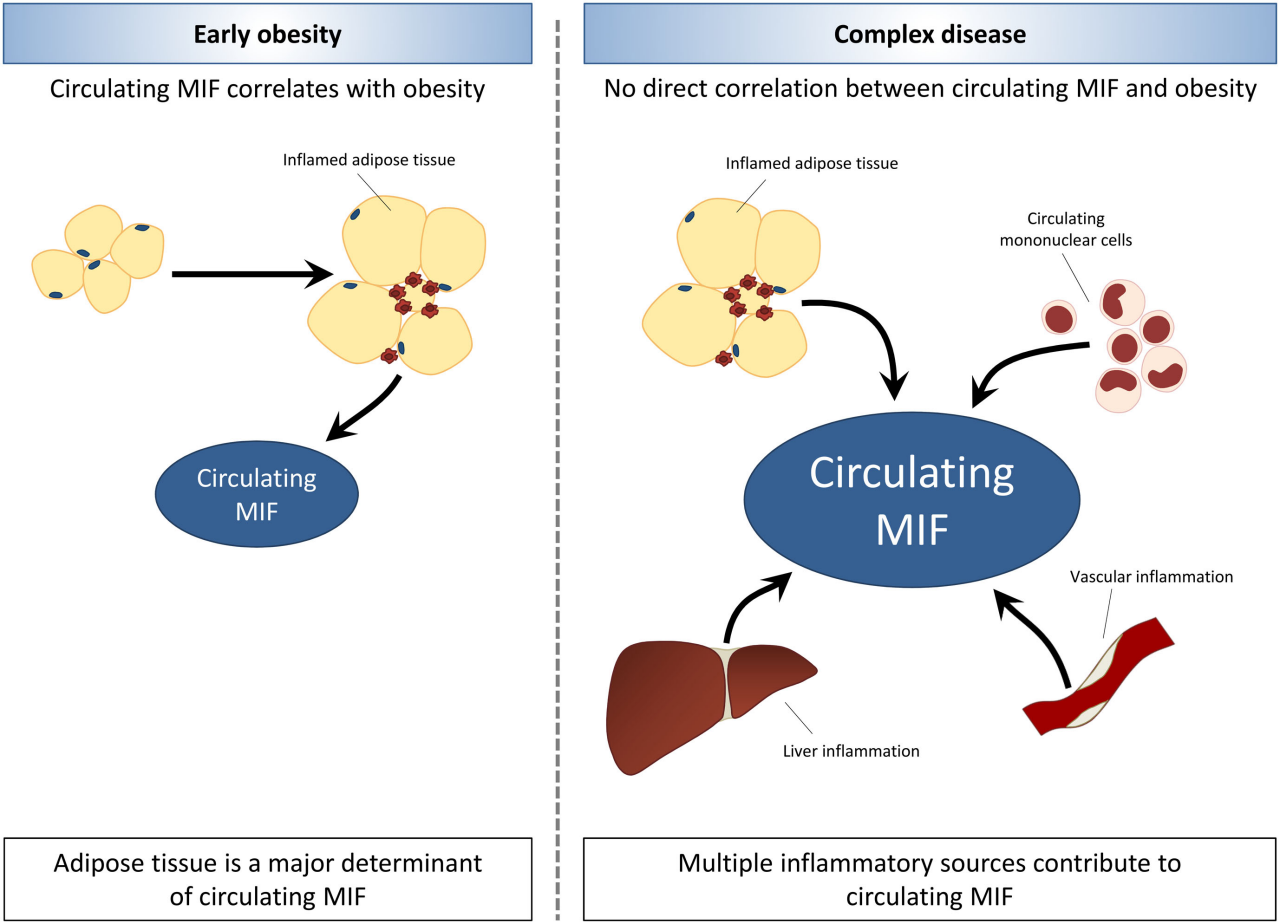
**Associations between MIF and obesity are lost with increasing disease complexity**. In early obesity, circulating MIF levels are increased in association with expansion and inflammation of adipose tissue. The development of obesity-related co-morbidities is related to elevated MIF levels, which rise with increasing severity of disease. In later stages of obesity-associated disease, MIF levels appear to be elevated independent of obesity and adiposity. Thus, indicating that other sources of inflammation such as circulating mononuclear cells, liver inflammation, and vascular inflammation may contribute to circulating MIF levels with increasing disease complexity.

Compared with the overweight/obesity studies, there are relatively few studies in insulin resistant/T2D subjects, and these have been conducted in very specific populations (e.g., Asians, Pima Indians). While there were intervention studies (weight loss, bariatric surgery) in overweight/obese subjects, no such reports exist in the case of T2D. Therefore, conclusions should be drawn carefully. Overall, circulating MIF seems to be associated with insulin resistance and overt T2D, and MIF levels rise with the severity of disease. This suggests that MIF-inducing processes unrelated to obesity/adiposity and adipose tissue expression may contribute to circulating MIF levels as overweight/obesity progresses to T2D. Inflammatory processes in other tissues or in circulating immune cells may be of importance in this respect. The involvement of sources of inflammation other than adipose tissue may explain that the associations of MIF and metabolic disease (metabolic syndrome, insulin resistance, T2D) appear to be independent of obesity with increasing complexity of disease (Figure 2).

### Relationship MIF and NAFLD

To date, only one study has examined the role of MIF in NALFD patients. Akyildiz et al. (56) investigated the effect of MIF-173 G/C gene polymorphism in NAFLD and found no difference in genotype or C-allele frequency between healthy controls (*n* = 104) and NAFLD patients (*n* = 91). Furthermore, they did not find any differences in genotype or C-allele frequency within the NAFLD patient group when subjects were stratified for NAFLD score (steatosis/borderline NASH/NASH) or fibrosis stage. Immunohistological investigation of MIF expression in liver biopsies from the NAFLD patients in this study showed that MIF staining was increased in both hepatocytes and MNCs in NASH patients compared to those with only steatosis. While hepatocyte MIF expression was not associated with fibrosis stage, fibrosis stage was significantly higher in presence of MIF staining in MNCs and positive MNC MIF staining was associated with a 3.6-fold increased risk of fibrosis.

## Animal Studies

Several studies in genetically engineered mouse models that lack the *Mif* gene have aimed to unravel the role of MIF in both non-pathological glucose homeostasis and in the obese/insulin resistant state. The results of these studies have been summarized in Table 3.

**Table 3:** **MIF in experimental models of obesity and insulin resistance/T2D**.

Model	Diet	Sex	Effect observed	Reference
*Mif*^−/−^ C57BL/6 mice	ns	Male	Glucose tolerance (ipGTT) is impaired in *Mif*-deficient mice.	(57)

*Mif*^−/−^ C57BL/6 mice	ns	Male	Insulin secretion after ipGTT is similar in *Mif*-deficient and wild-type mice (glucose response is not specified).	(58)

*Mif*^−/−^ C57BL/6 mice (from 129/Sv background)	ns	ns	Age-dependent impairment of glucose tolerance (ipGTT) in *Mif*-deficient mice.	(59)

Wistar rats	Standard diet + 10% fructose in drinking water	Male	Higher plasma MIF (tendency) in fructose-fed rats than controls. Correlation with visceral adipose tissue mass.	(60)

C57BL/6 mice	HFD (60% fat)	Male	Higher plasma MIF on HFD than on control diet.	(61)

*Mif*^−/−^ C57BL/6J mice	HFD (45 en% from palm oil)	Male	*Mif* deficiency protects against development insulin resistance: lower ipGTT and ipITT.	(62)

*Ldlr*^−/−^*Mif*^−/−^ mice (C57BL/6 background)	Chow	Male	*Mif* deficiency protects against development insulin resistance: fasting plasma glucose and insulin lower, improved ipGTT, ipITT, and euglycemic clamp.	(9)

*Mif*^−/−^ C57BL/6 mice	HFD (60 en% fat)	ns	*Mif* deficiency increases fasting glucose and impairs glucose tolerance (ipGTT).	(63)

*Mif*^−/−^ C57BL/6 mice (from *Mif*^−/+^ Balb/c background)	HFD (60% fat)	Male	*Mif* deficiency does not affect development of glucose intolerance (ipGTT) or plasma insulin levels.	(64)

STZ- and L-NAME-induced impaired glucose tolerance in Sprague-Dawley rats	Chow	ns	Plasma MIF levels higher in impaired glucose tolerance rats than in controls.	(48)

STZ-induced T2D in *Mif*^−/−^ Balb/c mice	ns	Female	*Mif*-deficient mice have lower STZ-induced blood glucose and better glucose tolerance (OGTT).	(49)
STZ-induced T2D in ICR mice + MIF antagonist (CPSI-1306)	ns	Female	MIF antagonism reduced STZ-induced blood glucose levels.	

C57BLKS/J db/db mice + MIF inhibitor (ISO-1)	Chow	Male	MIF inhibition normalized hyperglycemia and improved impaired glucose tolerance (ipGTT).	(65)

*ns, not specified*.

### Role of MIF in non-pathological glucose homeostasis

Nikolic et al. (57) demonstrated that *Mif*-deficient mice (male, 12 weeks old, diet not specified, C57BL/6 background) have impaired insulin signaling (AKT phosphorylation) in liver and adipose tissue. This was reflected in their response to an intraperitoneal glucose tolerance test (ipGTT), during which *Mif*^−/−^ mice had a similar insulin response to wild-type mice, but showed impaired clearance of glucose. In this experiment, glucocorticoid receptor inhibition rescued the observed impairment of insulin signaling in liver and adipose tissue, and enhanced glucose-stimulated insulin release and glucose clearance during the ipGTT. A second study by the same group (58) confirmed that *Mif*^−/−^ mice (male, 8–12 weeks old, diet not specified, C57BL/6 background) show a comparable insulin response to an ipGTT to wild-type mice (however, the glucose response was not shown in this study). A rationale for these observations was provided by results from *in vitro* experiments (58) that showed that conditioned medium containing insulin secreted from *Mif*^−/−^ pancreatic islets had lower activity than conditioned medium with insulin produced by wild-type islets, as demonstrated by reduced insulin signaling (AKT phosphorylation) and glucose uptake in a human liver carcinoma cell line (Hep G2 cells). Furthermore, this study revealed that MIF physically interacts and binds with insulin in wild-type islets and affects the conformation of insulin, reducing the concentration of monomer/dimer insulin and increasing hexamer formation of insulin, which may contribute to enabling insulin’s full functionality. These effects were found to be independent of MIF’s tautomerase activity.

Serre-Beinier and colleagues (59) investigated the role of MIF in carbohydrate homeostasis over time with aging. For this they used *Mif*^−/−^ C57BL/6 mice from a 129/Sv background (diet and sex not specified) and examined glucose and insulin tolerance over time. *Mif*^−/−^ mice had lower (13%) body weight at birth, but gained weight faster than wild-type mice. By 4 months of age, body weights were similar to those of wild-type mice, and at 12 months of age, they were 17% higher than in wild-type mice. While fasting blood glucose remained stable over time in both *Mif-*deficient and wild-type mice, fasting insulin increased over time in *Mif*^−/−^ mice but remained stable in wild-type mice. IpGTTs showed that *Mif*^−/−^ mice cleared glucose more rapidly than wild-type mice, but insulin levels were higher in the *Mif-*deficient mice, indicating that they required more insulin to achieve this effect. At 12 months of age, glucose clearance was impaired relative to wild-type mice, with comparable insulin levels. These results are in line with findings by Nikolic et al. (57) and Vujicic et al. (58) described above, which suggest that insulin produced in absence of MIF may have reduced activity/functionality. The response to exogenous insulin (during intraperitoneal insulin tolerance tests; ipITTs, and euglycemic–hyperinsulinemic clamps) was similar between genotypes at 4 and 12 months of age and there were no differences in insulin-stimulated AKT phosphorylation in muscle, indicating that insulin sensitivity of this peripheral tissue is not affected by MIF.

While these studies indicate that MIF may contribute to insulin function in non-pathological glucose homeostasis (i.e., in absence of obesity or disease), the associations observed in human studies indicate that it may have a detrimental role in obesity and the development of obesity-associated insulin resistance and T2D, i.e. conditions of metabolic stress and disturbed metabolic homeostasis. Several groups have addressed this using experimental models of obesity and insulin resistance/T2D.

### Role of MIF in (diet-induced) obesity and insulin resistance

Several groups have investigated the role of MIF in obesity and associated development of insulin resistance/T2D using high-fat diet (HFD)-induced rodent models of disease (Table 3). Velickovic and colleagues (60) reported higher plasma MIF levels in ­fructose-fed Wistar rats than in controls (13.6 ± 1.7 vs. 11.0 ± 0.9 ng/ ml), and showed that MIF levels correlated with visceral adipose tissue mass, consistent with observations made in humans. In line with this, Saksida et al. (61) showed that HFD feeding in C57BL/6 mice resulted in an obese phenotype, with increased fasting glycemia and serum insulin, which was accompanied by increased serum MIF levels (~7 ng/ml compared with ~0 ng/ml in control diet-fed mice). This study also showed that *Mif* mRNA expression in pancreatic islets was increased in HFD-fed mice. Furthermore, they showed that in wild-type pancreatic islets, palmitic acid (a saturated fatty acid which is present in human diets as well as in experimental HFDs) induces MIF production and apoptosis, while pancreatic islets from *Mif-*deficient mice were entirely resistant to palmitic acid-induced apoptosis, which may be of importance in β-cell apoptosis in late-stage obesity-associated T2D.

Finucane and colleagues (62) also described protective effects of *Mif* deficiency in HFD-induced obesity/insulin resistance. In this study, *Mif*^−/−^ mice (male, 8–9 weeks old, C57BL/6J background) were fed a palm oil based (rich in palmitic acid) HFD (45 kcal% fat) for 16 weeks. *Mif* deficiency reduced HFD-induced body weight gain and fat mass and improved the response to ipGTT and ipITT. Glucose clearance during the ipGTT was improved compared with wild-type mice, with a reduced insulin response and similarly, the *Mif*^−/−^ mice showed a more efficient glucose clearance in the ipITT. Together these glucose and insulin tolerance tests show that the response to both endogenous insulin (produced during ipGTT) and exogenous insulin (injected in ipITT) is improved in absence of MIF, thus suggesting an improvement in peripheral insulin signaling in obese *Mif*^−/−^ mice. These effects were also observed when mice were weight-matched to exclude effects of body weight differences, suggesting that *Mif* deficiency improves insulin signaling in peripheral tissues, such as white adipose tissue.

Support for this notion comes from studies that analyzed the effects of MIF on insulin signaling in adipose tissue. Finucane et al. (62) further showed that in wild-type mice, obesity increased MIF protein expression in the epididymal adipose tissue, for which the stromal vascular fraction rather than the adipocyte fraction was the primary cellular source. *Mif*^−/−^ mice were found to have reduced adipose tissue inflammation (reduced M1 macrophage infiltration and reduced *ex vivo* cytokine secretion) and this was accompanied by improved adipose tissue insulin signaling as demonstrated by increased insulin-stimulated AKT phosphorylation and *Glut4* expression *in vivo*, as well as increased insulin-stimulated glucose uptake into adipose tissue explants. Similarly, Verschuren et al. (9) used the hyperlipidemic *Ldlr*^−/−^ background to induce obesity and insulin resistance (male mice up to 52 weeks old, chow, C57BL/6 background) and showed that *Mif* deficiency protects against the development of insulin resistance. *Mif*-deficient mice had lower fasting glucose and insulin levels and showed improved glucose tolerance (assessed by ipGTT, with comparable insulin levels during the test) and improved insulin sensitivity (assessed by ipITT). Furthermore, a hyperinsulinemic–euglycemic clamp showed that insulin resistance was reduced in *Mif*-deficient mice. In line with the findings by Finucane et al. (62), analysis of adipose tissue in this study showed improvement of peripheral insulin signaling. This study also revealed improvements in adipose tissue inflammation in absence of MIF, with smaller adipocytes, reduced macrophage infiltration and crown-like structure formation and a lower M1/M2 macrophage ratio. This was accompanied by improved insulin signaling in adipose tissue (increased insulin-stimulated Pi3-Kinase activity and AKT phosphorylation), which further corroborates the observed improvements in glucose handling and insulin sensitivity. In both these studies, *Mif* deficiency improved the inflammatory milieu in adipose tissue and resulted in improvement of insulin signaling.

While above studies used HFDs with a moderate fat content (translational to the human situation), two studies that used diets with a supraphysiological fat content (60 kcal%), made different observations; Heinrichs et al. (63) reported that *Mif* deficiency in HFD-fed C57BL/6 mice (age and sex not specified) promotes weight gain and has detrimental effects on glucose tolerance (impaired glucose clearance during ipGTT). Remarkably, adipose tissue inflammation was reduced in absence of *Mif* in this study, in line with observations in the more physiological diet-induced obesity studies. A study by Conine and co-workers (64) showed no difference between *Mif*^−/−^ mice (male, 6–8 weeks old, C57BL/6 mice from Mif^−/+^ Balb/c background) and wild-type mice after 15 weeks of HFD feeding. In this study, body weight gain and adipose tissue inflammation were similar between genotypes, and there was no difference in glucose intolerance (assessed by ipGTT) or fasting blood glucose and insulin levels.

### Role of MIF in (STZ-induced) diabetes

Others have used streptozocin (STZ; a toxic glucose analog that preferentially accumulates in pancreatic beta cells but that can also be harmful to other tissues) to induce a (pre-)diabetic phenotype (severity depends on STZ dosage and regimen). Yu et al. (48) showed that plasma MIF levels were higher in a pre-diabetic rat model of impaired glucose tolerance (HFD-fed and STZ- and L-NAME-treated Sprague-Dawley rats) than in chow-fed controls (32.5 ± 1.9 vs. 24.8 ± 1.6 ng/ml). Sanchez-Zamora and co-workers (49) showed that *Mif* deficiency in Balb/c mice (female, age and diet not specified) lowered STZ-induced blood glucose levels and improved glucose tolerance in an oral glucose tolerance test (OGTT) without affecting insulin production by pancreatic β-cells. Similarly, MIF antagonism (with small molecule inhibitor CPSI-1306) reduced STZ-induced blood glucose levels in ICR outbred mice in the same study (49). Together, these studies in STZ-induced diabetes suggest an improvement of peripheral insulin sensitivity and/or more active insulin in *Mif*-deficient mice. The latter, however, would contradict findings by Nikolic et al. (57) and Vujicic et al. (58) discussed above.

Overall, these STZ studies lend further support to the notion that *Mif* deficiency protects from (pre)diabetes and are thus in line with most of the observations made in diet-induced models of obesity and metabolic disease. While in normal physiology, MIFs effects in glucose homeostasis were found to be independent of MIFs tautomerase activity (58), results from a study by Wang et al. (65) suggest that this activity may play a role under pathophysiological conditions. The authors showed that in diabetic db/db mice (male, 8 weeks old, chow, C57BLKS/J background), which have an autosomal recessive mutation in the leptin receptor, treatment with the MIF tautomerase activity inhibitor ISO-1 reduced fasting glucose levels to those observed in non-diabetic controls (db/m mice). These effects were further supported by results from an ipGTT, which revealed significant improvement in glucose handling in ISO-1 treated mice. In the lean, non-diabetic control mice, ISO-1 treatment did not affect fasting blood glucose levels or ipGTT responses, consistent with the discussed observations made by Vujijic et al. (58).

Altogether, these studies indicate that while MIF seems to have a function for insulin conformation and functionality in non-pathological glucose homeostasis, its role is less clear when metabolic stress and pathological processes come into play. Under these conditions, *Mif* deficiency appears to be protective against white adipose tissue inflammation and may improve glucose metabolism and peripheral insulin resistance although results are partly conflicting. An important explanation for these discrepancies is likely to be the choice of rodent model in combination with the experimental conditions employed. Inbred mouse strains are known to differ greatly in their susceptibility to obesity as well as in glucose metabolism and insulin resistance (66–68). Differences in the genetic background of knockout mice (as well as successfulness of backcrossing onto a different strain) are therefore likely to have a large impact on study outcomes. A second factor that may influence study results is the choice of diet (i.e., percentage of fat, type of fat, palmitic acid content) and the age that mice are started on this dietary treatment [for instance, older *Ldlr*^−/−^ mice show a more aggravated disease phenotype in response to HFD feeding than younger mice (69)]. Although information on the mouse strain and dietary conditions used are critical for the interpretation and comparison of different studies, they are often inadequately described. Overall, the prevailing finding is that *Mif* deficiency is protective in insulin resistance, glucose tolerance, and STZ-induced diabetes, and reduced adipose tissue inflammation and improved insulin signaling in adipose tissue may play a role in these beneficial effects, in line with observations made in human studies. Besides driving the development of insulin resistance and T2D in obesity, metabolic inflammation is also believed to drive the development of NAFLD (Figure 1).

### Role of MIF in non-alcoholic fatty liver disease

Several studies provide indication that MIF may also play a role in the development of hepatic steatosis, inflammation and fibrosis (Table 4). Finucane and colleagues (62) showed that *Mif* deficiency alleviates HFD-induced hepatic steatosis (male, 8–9 weeks old, 16 weeks HFD 45 kcal% fat from palm oil, C57BL/6J background). In *Mif*^−/−^ mice, liver weight was reduced, and hepatic triglyceride content (as well as histologically observed steatosis) was reduced. *Mif-*deficient mice also had lower plasma alanine transaminase (ALAT) levels. Hepatic gene expression analyses revealed that lipogenic gene expression (i.e., *Cd36*, *Dgat-1*, *Fasn*, *Srebp1-c*, *Pgc-a1*, *Lpl*, *Ppary*) was significantly lower in absence of MIF, providing a possible rationale for the observed reduction in hepatic steatosis. In line with these results, Heinrichs et al. (63) observed that hepatic steatosis was reduced in absence of MIF in both HFD-fed (60 kcal% fat, 16 weeks) and methionine and choline-deficient (MCD) diet-fed (8 weeks) *Mif*^−/−^ mice (sex and age not specified, C57BL/6 background). In the HFD-fed mice, *Mif* deficiency increased liver weight while liver damage markers ALAT and aspartate transaminase (ASAT) were slightly reduced. The observed reduction in hepatic steatosis (analyzed by histological Oil Red O staining and biochemical measurement of hepatic triglycerides) was accompanied by increased lipogenic gene expression (i.e., transcription factors *Lxra* and *Srebp-1*, and enzymes *Acc*, *Fas*, *Dgat1*, *Dgat2*), which was also observed in the MCD diet-fed mice. Furthermore, HFD-feeding resulted in increased hepatic inflammatory cell infiltration (1.6-fold increase in F4/80^+^ macrophages) in *Mif*^−/−^ mice, which were skewed toward a M2 polarization. Mechanistic *in vitro* experiments in oleic acid/IL-β stimulated mouse hepatoma cells Hepa1-6 and primary murine hepatocytes showed that recombinant MIF reduced hepatocyte lipid content through a CD74 and AMPK-mediated pathway. In contrast to the above studies, Conine et al. (64) observed no difference in HFD-induced (60 kcal% fat) hepatic lipid accumulation in presence or absence of *Mif* (male, 6–8 weeks old, C57BL/6 mice from *Mif*^−/+^ Balb/c background). It is not clear whether absence of phenotypical differences in this study may be related to the relatively young age of the mice at the start of the study, the extreme (supraphysiological) dietary fat content or the genetic background of the mice.

**Table 4:** **MIF in experimental models of NAFLD/liver fibrosis**.

Model	Diet	Sex	Effect observed	Reference
*Mif*^−/−^ C57BL/6J mice	HFD (45 en% from palm oil)	Male	Plasma ALT lower in *Mif*-deficient mice. Liver triglycerides, lipogenic gene expression, and pNFκB reduced in *Mif*-deficient mice.	(62)

*Mif*^−/−^ C57BL/6 mice	HFD (60 en% fat)	ns	*Mif* deficiency increases hepatic steatosis and hepatic immune-cell infiltration	(63)
	MCD diet	ns	Liver triglycerides increased in *Mif*-deficient mice.	

*Mif*^−/−^ C57BL/6 mice (from *Mif*^−/+^ Balb/c background)	HFD (60% fat)	Male	*Mif* deficiency does not affect hepatic lipid accumulation.	(64)

*Mif*^−/−^ C57BL/6 mice + CCl_4_	Chow (Teklad)	Male and female	Liver fibrosis in *Mif*-deficient mice similar to wild-type.	(70)

*Mif*^−/−^ C57BL/6 mice + CCl_4_ or + TAA	ns	ns	Liver fibrosis more severe in *Mif*-deficient mice (both CCL_4_- and TAA-induced).	(71)
C57BL/6 mice + CCl_4_ + rMIF	ns	ns	Treatment with rMIF reduced hepatic stellate cell activation and repressed expression of fibrosis-relevant genes.		

*ns, not specified*.

While the above studies focused mainly on the lipid accumulation that is observed in NAFLD, others have investigated the role of MIF in the development of hepatic fibrosis, a characteristic of progressive NASH. Barnes and co-workers (70) studied effects of MIF in chemically induced liver fibrosis (male and female, 10–12 weeks old, C57BL/6 *Mif*^−/−^ mice). In wild-type mice, a single dose of CCl_4_-induced mRNA expression of *Mif* and its cognate receptor *Cd74* (peaking between 4 and 8 h and resolving by 18 h), which was also observed in plasma with MIF protein levels increased as early as 2 h after injection (indicating a release of preformed pools of MIF). Circulating ALAT and ASAT were similarly increased in *Mif*^−/−^ and wild-type mice indicating comparative liver damage between genotypes. Assessment of hepatic stellate cell activation upon chronic CCl_4_ treatment (2 i.p. injections per week for 5 weeks) revealed increased mRNA expression of *Acta2* (α-SMA) and *Col1a1* (Collagen type I, alpha 1) in male *Mif*^−/−^ mice compared with wild-type, while expression of these genes was reduced in female *Mif*^−/−^ mice compared with wild-type mice. Despite these effects of *Mif* deficiency on hepatic stellate cell activation, hepatic collagen deposition (assessed by quantification of Sirius Red staining and hydroxyproline measurement) was comparable to wild-type mice in both male and female *Mif*^−/−^ mice. Authors then continued their studies in female mice only, to investigate a potential role of MIF on the progression of fibrosis via the regulation of extracellular matrix (ECM) degradation. They observed reduced infiltration of restorative macrophages in *Mif*^−/−^ mice, which was accompanied by reduced expression (mRNA, protein, and enzymatic activity) of the ECM-degrading metalloproteinase MMP13, suggesting a possible proresolution role for MIF during fibrosis.

Heinrichs and colleagues (71) also investigated the effects of *Mif* deficiency (age and sex not specified, C57BL/6 *Mif*^−/−^ mice) in two different models of chemically induced liver fibrosis. In this study, hepatic fibrosis (assessed histologically and by hydroxyproline measurement) was increased in absence of MIF in both CCl_4_- and TAA-induced liver fibrosis. In line with this, *Mif*-deficient mice had increased mRNA expression of *Col1a1*, *Timp1*, *Mmp2*, and *Tgfb1*, as well as increased α-SMA protein expression. FACS analysis revealed no differences between genotypes in immune-cell subset infiltration, suggesting that differences in fibrosis development may be explained by effects on hepatic stellate cell activation rather than by effects on the intrahepatic immune response. *In vitro* studies in murine hepatic stellate cells showed that treatment with recombinant MIF inhibited PDGF-induced hepatic stellate cell activation, which was found to be mediated through CD74 and AMPK signaling. In line with these *in vitro* results, follow-up *in vivo* studies showed that CCl_4_-induced hepatic fibrosis was increased in *Cd74*^−/−^ mice. Deficiency of *Cxcr4*, however, another MIF receptor expressed by hepatic stellate cells, did not affect fibrosis development. Therapeutic application of recombinant MIF in CCl_4_-treated wild-type mice reduced hepatic stellate cell activation and repressed expression of fibrosis-relevant genes. However, the effects of this recombinant MIF treatment on fibrosis development are not mentioned.

Overall, very few studies to date have investigated the effects of MIF in the development of hepatic co-morbidities. MIF appears to have detrimental effects in HFD-induced hepatic lipid accumulation through effects on lipogenic gene expression, providing further support for the notion that MIF may indeed not only have pro-inflammatory effects but may also be linked to metabolism. In chemically-induced liver fibrosis, it seems that MIF may have protective effects, possibly through an effect on the resolution of fibrosis. However, results from a clinical study in NAFLD patients indicate the opposite, showing that increased MIF expression in liver is associated with increased risk of fibrosis. This discrepancy may be explained by differences in disease etiology in chemically induced liver damage used in the experimental disease models, compared with the metabolically induced liver damage that is typical for human NAFLD development.

## Conclusion and Future Directions

In early obesity, circulating MIF levels are increased in association with expansion and inflammation of adipose tissue. The development of insulin resistance and T2D in obesity is related to elevated MIF levels, which rise with increasing severity of disease. In later stages of obesity-associated T2D, MIF levels appear to be elevated independent of obesity and adiposity, indicating that with increasing disease complexity, other sources of inflammation may contribute to circulating MIF levels.

Mechanistic studies in experimental models of disease have shown that MIF contributes to insulin function in non-pathological glucose homeostasis (i.e., in absence of obesity or disease) but its role is less clear when metabolic stress and pathological processes come into play. Under these conditions, *Mif* deficiency appears to be protective against adipose tissue inflammation and may improve insulin resistance and T2D in both HFD- and STZ-induced models. Furthermore, there are some first indications that MIF may contribute to development of diet-induced hepatic steatosis in NAFLD, while observations in advanced liver disease are conflicting, describing both pro- and anti-fibrotic effects of MIF.

To fully understand the intricacies of MIFs contribution to obesity-related disease development, future work is needed to decipher temporal (non-pathological – early disease – advanced complex disease) and spatial (tissue-specific) effects of MIF, as well as interorgan crosstalk that may be mediated by circulating MIF. For the interpretation of such studies, it is of paramount importance that they would be performed in translational, physiological (diet-induced) models under well-described experimental conditions. In addition, future studies assessing the complex interplay between metabolic and inflammatory balances will greatly enhance our understanding of the development of obesity-related diseases, and the role of MIF therein. Although most published data on MIF point toward a detrimental role in advanced stages of disease, the potential protective effects of this mediator under certain conditions deserve further investigation.

## Author Contributions

MM and RK had substantial contributions to the conception and design of the work, drafted and revised the manuscript, had final approval of the version to be published and agree to be accountable for all aspects of the work.

## Conflict of Interest Statement

The authors declare that the research was conducted in the absence of any commercial or financial relationships that could be construed as a potential conflict of interest.

## Abbreviations

ALAT, alanine transaminase; ASAT, aspartate transaminase; HFD, high-fat diet; HOMA, homeostatic model assessment; ipGTT, intraperitoneal glucose tolerance test; ipITT, intraperitoneal insulin tolerance test; MCD, methionine and choline deficient; MIF, macrophage migration inhibitory factor; MNC, mononuclear cell; NAFLD, non-alcoholic fatty liver disease; NASH, non-alcoholic steatohepatitis; T2D, type 2 diabetes.
